# An epidemiological survey of occupational stress among teachers in Tunisia

**DOI:** 10.1192/j.eurpsy.2025.1558

**Published:** 2025-08-26

**Authors:** A. Aloui, M. Bouhoula, E. Toulgui, N. Ganoun, A. Chouchane, M. Maoua, A. Brahem, H. Kalboussi, W. Ouanes, O. El Maalel, S. Jemni, I. Kacem, S. Chatti

**Affiliations:** 1 Occupational Medecine Department, University Hospital of Farhat Hached, Sousse, Tunisia; 2 University Of sousse, Faculty of Medecine Ibn El Jazzar, 4000 Sousse, Tunisia; 3 Research laboratory LR 19SP03, Study of the risks and prospects for the prevention of non-communicable diseases in the workplace; 4 Physical Medecine and Rehabilitation, University Hospital of Sahloul, Sousse, Tunisia, Tunisia

## Abstract

**Introduction:**

Stress is one of the biggest problems facing teachers today. The increasingly demanding nature of their job has also increased pressure levels dramatically. Research shows that teachers are now facing greater day-to-day problems with occupational stress than most other employees.

**Objectives:**

Determine the prevalence and the caracteristics of the occupational stress among teachers.

**Methods:**

This is an exhaustive cross-sectional study carried out among the teachers of a private teaching group in the city of Sousse in Tunisia between October and December 2022. The data were collected using an anonymous questionnaire in electronic format ‘Microsoft Forms’ distributed by e-mail. The questionnaire included socio-professional and medical data. Ocuupational stress was assessed using the Karasek questionnaire.

**Results:**

A total of 241 questionnaires were included, representing a response rate of 90.14%. The mean age of the teachers in this study was 39.98 ± 9.14 years, with a predominance of females (56.8%) and a sex ratio (M/F) of 0.75. In this private teaching group, there were 181 higer education teachers (75.1%) and 60 school teachers (24.9%).The average seniorty in this educational group was 3.49 ± 2.95 years. Participants worked an average of 4.88 ± 1.49 hours per day and 16.92 ± 7.39 hours per week. The majority of respondents had no medical (82.6%) or surgical (71.8%) co-morbidities. Only 10 (4.1%) teachers were anaemic, 6 (2.5%) were hypertensive and 4 (1.7%) were diabetic. More than half (55.6%) of the teachers had a BMI above the normal range: 102 participants were overweight (42.3%) and 32 (13.3%) were obese. As for lifestyle habits, 12.9% of teachers were smokers, 9.5% drank alcohol and 34.4% practised a sporting activity. Referring to Karasek’s two-dimensional model, 59 (24.5%) of the 241 teachers were classified in a situation of <<Jobstrain>>associating: low latitude and high professional demands. By completing this model with the third dimension of social support at work, 35 (14.5%) of the 241 teachers were classified in a situation of <<Isostrain>> combining low latitude, high professional demands and low social support.

**Conclusions:**

Action to improve work organisation and support for teachers would be necessary to prevent the occupational stress.

**Disclosure of Interest:**

None Declared

